# Characterization of the humoral and cellular immunity induced by a recombinant BCG vaccine for the respiratory syncytial virus in healthy adults

**DOI:** 10.3389/fimmu.2023.1215893

**Published:** 2023-07-18

**Authors:** Gaspar A. Pacheco, Catalina A. Andrade, Nicolás M.S. Gálvez, Yaneisi Vázquez, Linmar Rodríguez-Guilarte, Katia Abarca, Pablo A. González, Susan M. Bueno, Alexis M. Kalergis

**Affiliations:** ^1^ Millennium Institute on Immunology and Immunotherapy, Facultad de Ciencias Biológicas, Pontificia Universidad Católica de Chile, Santiago, Chile; ^2^ Millennium Institute on Immunology and Immunotherapy, Facultad de Medicina, Pontificia Universidad Católica de Chile, Santiago, Chile; ^3^ Departamento de Enfermedades Infecciosas e Inmunología Pediá trica, División de Pediatría, Escuela de Medicina, Pontificia Universidad Católica de Chile, Santiago, Chile; ^4^ Departamento de Endocrinología, Facultad de Medicina, Pontificia Universidad Católica de Chile, Santiago, Chile

**Keywords:** hRSV, vaccine, BCG, clinical trial, cellular response

## Abstract

**Introduction:**

The human respiratory syncytial virus (hRSV) is responsible for most respiratory tract infections in infants. Even though currently there are no approved hRSV vaccines for newborns or infants, several candidates are being developed. rBCG-N-hRSV is a vaccine candidate previously shown to be safe in a phase I clinical trial in adults (clinicaltrials.gov identifier #NCT03213405). Here, secondary immunogenicity analyses were performed on these samples.

**Methods:**

PBMCs isolated from immunized volunteers were stimulated with hRSV or mycobacterial antigens to evaluate cytokines and cytotoxic T cell-derived molecules and the expansion of memory T cell subsets. Complement C1q binding and IgG subclass composition of serum antibodies were assessed.

**Results:**

Compared to levels detected prior to vaccination, perforin-, granzyme B-, and IFN-γ-producing PBMCs responding to stimulus increased after immunization, along with their effector memory response. N-hRSV- and mycobacterial-specific antibodies from rBCG-N-hRSV-immunized subjects bound C1q.

**Conclusion:**

Immunization with rBCG-N-hRSV induces cellular and humoral immune responses, supporting that rBCG-N-hRSV is immunogenic and safe in healthy individuals.

**Clinical trial registration:**

https://classic.clinicaltrials.gov/ct2/show/, identifier NCT03213405.

## 1 Introduction

The human respiratory syncytial virus (hRSV) is the leading etiological agent for acute lower respiratory tract infections (ALRTIs) in children under five years old, geriatric, and immunocompromised populations (1). Worldwide, this virus is responsible for over 30 million hospitalizations and over 200,000 deaths annually (1, 2). Symptoms of ALRTIs include nasal congestion, fever, wheezing, bronchoconstriction, bronchial and alveolar collapse due to excessive mucus, and can lead to pneumonia (3). However, symptoms related to hRSV infection may go beyond acute disease, as evidence shows that infection in infants may lead to asthma and neurological or cognitive impairment (4, 5). Most vaccine candidates that are being developed are based on the use of the fusion (F) protein of hRSV (6). This year, the FDA approved two vaccines against hRSV for the geriatric population only, which are based on the stabilized pre-fusion protein of hRSV (7). However, there are no licensed hRSV vaccines for newborns or infants to date, despite the substantial social and economic burden that hRSV poses in this population.

One of the hRSV vaccine candidates is the vaccine prototype rBCG-N-hRSV, which is based on a *Mycobacterium bovis* Bacillus Calmette-Guérin (BCG) platform expressing the gene of the hRSV nucleoprotein (N-hRSV) (8, 9). Importantly, BCG is one of the oldest and safest vaccines used in the world, commonly applied to newborns to prevent tuberculosis in many countries worldwide and induces generalized T_H_1 immune responses. Thus, using BCG as a vector for an hRSV vaccine provides significant advantages (10). Moreover, this BCG strain could protect against both tuberculosis and hRSV in infants under six months old, the most at-risk population of hRSV-induced ALRTIs. Importantly, the N protein of hRSV was chosen as the viral protein to be expressed in the recombinant BCG because it is highly conserved throughout different hRSV isolates and because this protein modulates T cell activation by antigen-presenting cells (11, 12). rBCG-N-hRSV has been extensively tested in animal models and has been shown to promote protective humoral and cellular immunity against hRSV after viral challenge in both mice and calves (8, 13–15).

A randomized, double-blind, dose-escalating phase I clinical trial was held in Chile in 2017-2018 to evaluate the safety, tolerability, dissemination, and shedding of this live attenuated vaccine candidate in male adults (9). During this study, two participants per cohort were immunized with 2x10^5^ CFU of BCG-WT, and six participants were immunized with escalating doses of rBCG-N-hRSV. Each of the three cohorts was sequentially immunized (after approval by a DSMB), reaching a final dose of 1x10^5^ CFU of rBCG-N-hRSV. The vaccine was found to be safe and well-tolerated in adults at all doses tested, and no serious adverse events attributed to the vaccine were found. No evidence of dissemination or shedding of this BCG strain was found (9). Preliminary immunogenicity analyses showed that rBCG-N-hRSV induced a CD4^+^ T cell-driven cellular immune response characterized by enhanced IFN-γ and IL-2 secretion after stimulation with either N-hRSV or mycobacterial antigens, especially at higher doses (9). Total IgG against N-hRSV or mycobacterial antigens was not found to be statistically increased after immunization. These data are consistent with previous results from other studies using BCG as a vehicle for different vaccine candidates, showing a significant cellular immune response, yet a modest humoral immune response (16). Remarkably, neutralizing antibodies increased in two subjects from the cohort immunized during the peak hRSV season. This increase in neutralizing antibodies could arise from natural exposure to hRSV, but this possibility was not previously evaluated. Since no licensed vaccines against hRSV are currently approved, and the licensing of a vaccine for the pediatric population is mandatory, we sought further evaluation of the immune response induced against hRSV and mycobacterial antigens in samples from this phase I clinical trial for this rBCG-N-hRSV candidate vaccine as it advances into a phase II trial.

A deeper characterization of the immune response elicited by this vaccine is also relevant to rule out potentially excessive inflammation induced by the vaccine, as well as the functional capabilities of the antibodies it induces. This is important because non-neutralizing antibodies could eventually promote complement deposition in the lungs leading to subsequent lung damage (17). Similarly, the characteristics of the cellular immunity elicited by the vaccine are poorly understood. Furthermore, learning more about the cellular function could provide insights into potential CD8^+^ T cell-driven cytotoxic responses that may be promoted by immunization with this vaccine candidate. Here, we further dissected the cellular and humoral immune response elicited by this promising vaccine candidate as an extension and more in-depth analysis of the samples obtained from the phase I clinical trial described previously (9).

## 2 Methods

### 2.1 Phase I clinical trial study design and sample collection

Although an initial evaluation of the rBCG-N-hRSV vaccine candidate was performed in a phase I clinical trial (clinicaltrials.gov identifier #NCT03213405), we sought to perform a deeper immunogenicity analysis to further evaluate this vaccine candidate. Briefly, a randomized, double-blind, dose-escalating phase I clinical trial was designed and carried out between 2017-2018 in Santiago, Chile (NCT03213405). The primary and secondary outcomes were to evaluate the safety, tolerability, dissemination, shedding, and immunogenicity of the rBCG-N-hRSV vaccine in healthy adults. The protocol of this study followed the current ethical guidelines, such as Tripartite Guidelines for Good Clinical Practices, the Declaration of Helsinki (18), and local regulations. This study was approved by both the Institutional Ethical Committee (number 15216) and the Chilean Public Health Institute (ISP Chile, number EC819077/16). Inclusion and exclusion criteria and more information about the clinical trial can be found in the initial publication describing this clinical trial, which evaluated safety and tolerability (9).

During the study period, hRSV circulation was reported in Chile, as previously described (9). Particularly, the immunization of the subjects in Cohort A was performed during the peak of hRSV, Cohort B during the end of the hRSV season, and Cohort C during a period of low circulation of hRSV (9). Considering the immunization timeline, seroconversion analyses were performed to determine possible exposure to hRSV from vaccinated volunteers, as indicated in the results section. Further information regarding the outcomes, the ethical approval, inclusion and exclusion criteria, and the recruitment of volunteers can be found in the **Supplementary Information** (SI) previously published in (9). Briefly, 24 male adults aged 18-50 years were enrolled and randomized into three cohorts of eight subjects. In each cohort, two participants were immunized intradermally in the deltoid area with 2x10^5^ CFU of BCG-WT (Moscow strain). Since the population in Chile was immunized at that time with the Moscow BCG strain at birth, it was used as BCG-WT during this trial. The remaining six subjects were vaccinated with escalating doses of rBCG-N-hRSV (5x10^3^, 5x10^4^, or 1x10^5^ CFU), which were generated using the BCG Danish strain. The dose of BCG-WT selected is the dose most commonly used in newborns worldwide, and the doses of rBCG-N-hRSV used were escalating doses previously evaluated during pre-clinical studies and shown to be appropriate. Data obtained from subjects immunized with BCG-WT were analyzed together, independently of their cohort. Blood samples were taken before immunization (Day 0), as well as 14-, 30-, 60-, 120-, and 180-days post-immunization. Peripheral blood mononuclear cells (PBMCs) and sera were isolated from these blood samples and stored in liquid nitrogen and at -80°C, respectively, until use.

### 2.2 Perforin/granzyme B ELISPOT assays

Secretion of perforin (Perf) and granzyme B (GrzB) by PBMCs was evaluated using the Human Granzyme B/Perforin Double-Color Enzymatic ELISPOT Assay (ImmunoSpot^®^). The assay was performed according to the instructions given by the manufacturer, incubating with each stimulus for 48 h at 37°C and 5% CO_2_. 2x10^5^ PBMCs were plated and stimulated with either 1.25 µg/mL of N-hRSV (Genscript) or 750 IU/mL of bovine PPD (ThermoScientific) diluted in sterile RPMI 1640 medium (Gibco) supplemented with 2 g/L sodium bicarbonate, 10% FBS (Biological Industries), 2 mM L-Glutamine (Gibco), 0.2% v/v β-mercaptoethanol, and 1X Anti-Anti (Gibco), for 48 h at 37°C and 5% CO_2_. Stimulation of PBMCs with 5 µg/mL ConA or 0.5% sterile PBS were used as positive and negative stimulation internal controls, respectively (data not shown). After developing, plates were air-dried face-down on a paper towel for 24 h, and then stored with the underdrain on until spots were counted with an ImmunoSpot S6 CORE Analyzer (Immunospot).

### 2.3 Cytokine measurements in sera by Cytometric Bead Array™

Cytokine measurements were performed via the BD™ Cytometric Bead Array (CBA) Human Th1/Th2/Th17 Cytokine Kit (BD Biosciences). Determinations were performed following the instructions of the manufacturer. Briefly, once mixed and equilibrated, beads were mixed with either undiluted sera or PBMC culture supernatant diluted 1:4 in dilution buffer. Samples were acquired in an LSRFortessa X-20 flow cytometer (BD Biosciences). The median fluorescent intensity of PE in each bead population was calculated for every sample. A 4-parameter logistic (4PL) standard curve was constructed using the median fluorescent intensity of PE for each cytokine, subtracting the signal from the blank tube to each sample and standard and constraining the curve with a Bottom parameter = 0. Cytokine concentration was then interpolated in the respective standard curve.

### 2.4 Flow cytometry for T cell identification

PBMCs stored in liquid nitrogen were thawed, stimulated with antigens, and analyzed as previously described (9). Data were acquired in a BD LSRFortessa X-20™. Gating and analysis were performed using FlowJo software (V10.6.2).

### 2.5 C1q binding assay

The binding of C1q was measured using ELISA. Plates were coated overnight at 4°C with 50 µL of either 1 µg/mL N-hRSV (Genscript) or 25 µg/mL bovine PPD (ThermoScientific) in 100 mM bicarbonate/carbonate pH 9.5 buffer. Then, plates were washed three times with 200 µL of PBS-Tween 20 0.05% (Wash buffer) and then blocked for two h with 200 µL of PBS-Milk 5% (Blocking solution). Sera samples were diluted 1:30 in Blocking solution and inactivated at 56°C for 30 min. Plates were washed three times, incubated with 50 µL of inactivated sera samples for 1 h at RT, washed three times, incubated for one h with 50 µL of 4 µg/mL purified human C1q (Sigma-Aldrich) diluted in Blocking solution, washed five times, incubated for one h with 50 µL of mouse anti-human C1q (Invitrogen) diluted 1:2,000, washed seven times, incubated for two h with 50 µL of rat anti-mouse IgG1-HRP (Invitrogen) diluted 1:2,000, washed seven times with 200 µL of Wash buffer and washed manually one time with 200 µL of PBS. 50 µL of TMB (ThermoScientific) was added and incubated for 15 min at RT. Then, 50 µL of 2 N sulfuric acid was added and absorbance was immediately measured at 450 nm. A C1q standard curve was incorporated in every plate. To normalize for variable antibody concentration, a C1q Binding Index was defined and calculated as follows:


C1q Binding Index= Bound C1q (ng) / Log10(Antibody Concentration [ng/mL])

### 2.6 ELISA for IgG quantification

ELISA plates were coated overnight. at 4°C with 50 µL of 2 µg/mL N-hRSV (Genscript) or 25 µg/mL bovine PPD (Thermo Scientific) in sodium carbonate/bicarbonate 100 mM pH 9.5 buffer. Then, plates were washed three times with 200 µL of PBS-Tween 20 0.05% (Wash buffer). Plates were blocked with PBS-FBS 10% (Blocking solution for IgG1 and IgG2) or PBS-milk 5% (Blocking solution for IgG3) for 2 h at RT, washed three times, incubated with sera diluted 1:5 in Blocking solution for two h (IgG1 and IgG2) or 1:100 (IgG3), washed three times, incubated for 1 h with 50 µL of either rat anti-human IgG1 (BioLegend), rat anti-human IgG2 (BioLegend) or rat anti-human IgG3 diluted 1:500, washed three times, incubated for 1 h with 50 µL of rat anti-mouse IgG1-HRP (Invitrogen) diluted 1:2,000, washed three times with 200 µL of Wash buffer and manually washed one time with 200 µL of PBS. 50 µL of TMB Substrate Reagent (Thermo Scientific) was added to each well and developed for 15 min at RT. 50 µL of 2 N sulfuric acid was added to each well and absorbance at 450 nm was immediately measured. Standard curves for IgG1 or IgG2 were incorporated for every plate.

### 2.7 Virus neutralization tests

The evaluation of neutralizing capacity of the antibodies induced upon immunization was measured as previously described (9). Briefly, HEp-2 cells were incubated with 100 plate-forming units (PFU) of a recombinant GFP-hRSV virus. Then, inactivated sera samples were diluted, incubated with the infected cells for 1 h, and then replaced with fresh media (MEM). After incubation for 48 h, GFP^+^ PFUs were visualized and quantified using an epifluorescence microscope. Further details can be found in the previous report (9).

### 2.8 Anti-F-hRSV and anti-G-hRSV ELISAs for screening of hRSV exposure

ELISA plates were coated overnight at 4°C with 50 µL of 1 µg/mL F-hRSV (A2) (Sino Biological) or G-hRSV (B1) (Sino Biological) in sodium carbonate/bicarbonate 100 mM pH 9.5 buffer. Then, plates were washed three times with 200 µL of PBS-Tween 20 0.05% (Wash buffer). Plates were blocked with PBS-milk 5% (Blocking solution) for 1 h at RT, washed three times, incubated with sera diluted 1:5 in Blocking solution for 2 h, washed three times, and incubated for one h with 50 µL of sera samples serially diluted ranging from 1:100 to 1:3,200 in blocking solution. Then, plates were washed three times, incubated for 1 h with 50 µL of rat anti-human IgG-HRP (Thermo Scientific) diluted 1:2,000 in blocking solution, washed five times with 200 µL of Wash buffer and manually washed one time with 200 µL of PBS. 50 µL of TMB Substrate Reagent (Thermo Scientific) was added to each well and developed for 10 min at RT. 50 µL of 2** N** sulfuric acid was added to each well and absorbance at 450 nm was immediately measured.

### 2.9 Statistical analyses

All graphs and statistical analyses were performed in GraphPad Prism, version 9.1.0. Analyses of standard curves in ELISA assays were performed by a 4-parameter logistic regression (4PL) curve, constrained to a Bottom parameter = 0. Data were then interpolated in those regressions. To calculate fold changes relative to pre-immune conditions, the observed response variable at a given time was divided by that observed at Day 0. A two-way ANOVA for repeated measures with *post-hoc* Dunnett's test corrected for multiple comparisons against Day 0 was performed to evaluate differences after immunization. Base 10 logarithms of fold changes were calculated before statistical analyses.

## 3 Results

### 3.1 rBCG-N-hRSV elicits both hRSV- and mycobacterial-specific cytotoxic cell responses

One of the advantages of rBCG-N-hRSV, relative to other hRSV vaccine candidates, is the potential induction of both, anti-mycobacterial and anti-hRSV immune responses (8). While CD4^+^ helper T cell responses against N-hRSV and mycobacterial antigens after rBCG-N-hRSV immunization have been previously evaluated (9), the secretion of cytotoxic molecules by CD8^+^ cytotoxic T cells has yet to be assessed. Importantly, a robust CD8^+^ T cell response correlates with protection against hRSV in humans (19), so the induction of an effector CD8^+^ T cell response would be desirable for a vaccine against this virus.

**Figure 1:**
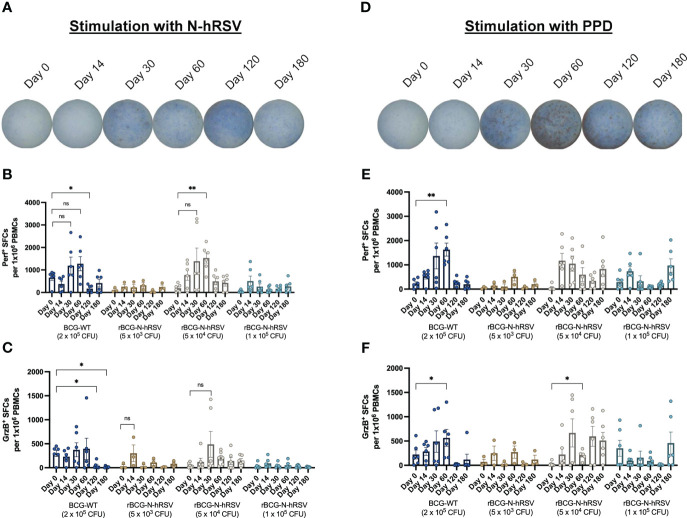
PBMCs from subjects immunized with 5x10^4^ CFU of rBCG-N-hRSV show enhanced Perf and GrzB production upon stimulation of with N-hRSV or PPD. Representative images of wells in which PBMCs from subjects immunized with 5x10^4^ CFU of rBCG-N-hRSV were stimulated with either **(A)** N-hRSV or **(B)** PPD. Images are representative of subjects immunized with 5x10^4^ CFU of rBCG-N-hRSV. Blue spots are Perf^+^ spot-forming cells (SFCs), and red spots are GrzB^+^ SFCs. **(B, E)** Perf^+^ SFCs and **(C, F)** GrzB^+^ SFCs were counted after PBMCs were stimulated for 48 h with either 1.25 µg/mL of N-hRSV **(B, C)** or 750 IU/mL of PPD **(E, F)**. Stimulation of PBMCs with 5 µg/mL ConA or 0.5% sterile PBS were used as positive and negative stimulation controls, respectively (data not shown). The concentration of the stimulus was selected according to the purpose of the evaluation to be performed. Data for subjects potentially exposed to hRSV during the post-immunization period, as suggested by hRSV-specific serological assays, were excluded. Bars represent the mean value of SFCs, and error bars represent the SEM. A two-way ANOVA for repeated measures with *post-hoc* Dunnett's test corrected for multiple comparisons against Day 0 was performed for data analysis. ns= not significant, * = P<0.05, ** = P<0.01.

Stimulation of PBMCs with N-hRSV demonstrated a trend to induce an effector cytotoxic molecule response in subjects immunized with 5x10^4^ CFU of rBCG-N-hRSV (**Figure 1**, **Supplementary Figures 1**, **2**). A progressive increase of perforin (Perf) and granzyme B (GrzB) response were observed in PBMCs from subjects of this cohort, with a significant increase in Perf secretion at day 60 post-immunization (**Figure 1**). It must be considered that the results of the GrzB response from subjects of this cohort showed high variability between subjects at day 30 post-immunization. Interestingly, the GrzB response obtained with 5x10^4^ CFU was similar to 5x10^3^ CFU of rBCG-N-hRSV (**Figure 1**). Lastly, immunization with BCG-WT induced a stable secretion of cytotoxic molecules in PBMCs in response to N-hRSV during 30- and 60-days post-immunization, yet not significant.

Stimulation of PBMCs with PPD antigen induced an effector cytotoxic molecule response in subjects immunized with 5x10^4^ CFU of rBCG-N-hRSV, as compared to subjects immunized with other rBCG-N-hRSV doses (**Figure 1**). However, this response was modest as compared to subjects immunized with 2x10^5^ CFU of BCG-WT. Subjects vaccinated with BCG-WT showed a progressive increase in responsiveness against PPD after immunization (**Figure 1**). Subjects immunized with 5x10^3^ CFU of rBCG-N-hRSV showed a low response to PPD, probably due to the low dose of rBCG-N-hRSV in this cohort. Subjects that received 1x10^5^ CFU of rBCG-N-hRSV showed an optimal response to PPD, but lower as compared to subjects immunized with 5x10^4^ CFU of rBCG-N-hRSV (**Figure 1**). Even though the inhibition of the PPD antigen expression by the recombinant N-hRSV is highly unlikely since mycobacteria constitutively express PPD antigens, it needs to be taken into consideration that the strain used in the dose of BCG-WT and rBCG-N-hRSV are different, and therefore the response to PPD should not be expected to be the same.

These results suggest that the trend to increase the secretion of Perf and GrzB in response to N-hRSV stimulation depends on the amount of N-hRSV in the corresponding rBCG dose (**Figure 1**). Therefore, among the rBCG doses, the 5x10^4^ CFU of rBCG-N-hRSV seems like an optimal dose for inducing the secretion of cytotoxic molecules by PBMCs in response to either PPD or N-hRSV.

### 3.2 rBCG-N-hRSV immunization triggers cytokine secretion by PBMCs stimulated with hRSV or mycobacterial antigens

**Figure 2:**
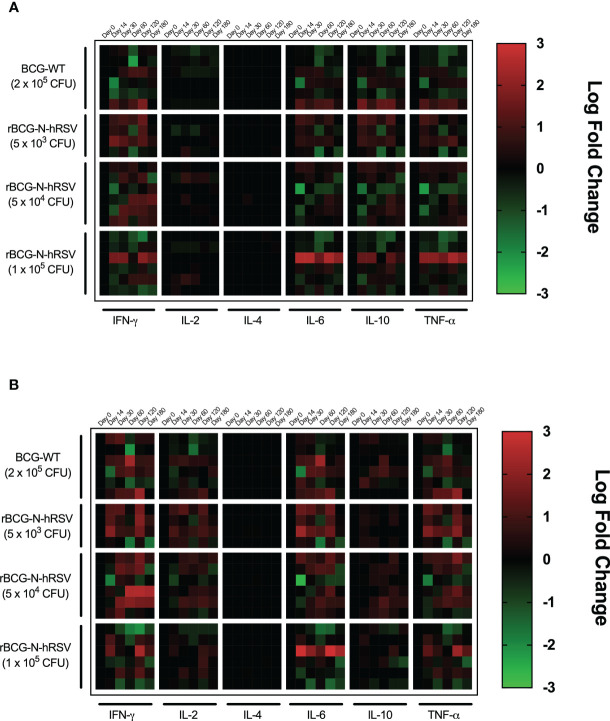
PBMCs from subjects immunized with 5x10^3^ or 5x10^4^ CFU of rBCG-N-hRSV produce IFN-γ upon stimulation with N-hRSV and PPD. Heatmap of log_10_ fold change of the concentration of the cytokines IFN-γ, IL-2, IL-4, IL-6, IL-10, and TNF-α in supernatants of PBMC cultures stimulated with **(A)** 1.25 µg/mL N-hRSV or with **(B)** 750 IU/mL PPD respect to day 0. Each block of columns represents a particular cytokine, labeled below. Individual columns represent time points after immunization, specified above. Each block of rows represents one specific cohort of immunized study subjects labeled left. Individual rows represent individual subjects. A color scale is depicted on the right. Data for subjects potentially exposed to hRSV during the post-immunization period, as suggested by hRSV-specific serological assays, were excluded.

The secretion of various cytokines by PBMCs after stimulation with N-hRSV or PPD (**Figure 2**) was measured to assess changes in pro- or anti-inflammatory cytokines after immunization. Stimulation of PBMCs with N-hRSV led to increased levels of IFN-γ, IL-6, IL-10, and TNF-α after immunization with 5x10^3^, 5x10^4^, and 1x10^5^ CFU of rBCG-N-hRSV (**Figure 2**). Subjects immunized with BCG-WT also showed an upregulation of the same cytokines, still the increase was observed on a single subject. Stimulation with PPD led to increased levels of IFN-γ, TNF-α, IL-2, IL-6, and IL-10 (**Figure 2**) in subjects immunized with 5x10^3^ or 5x10^4^ CFU of rBCG-N-hRSV. Subjects immunized with 2x10^5^ CFU of BCG-WT also showed an upregulation of IL-6.

This suggests that immunization with either 5x10^3^ or 5x10^4^ CFU of rBCG-N-hRSV leads to the secretion of pro-inflammatory cytokines typically associated with immune responses against intracellular pathogens, such as viruses or mycobacteria (20–22).

Additionally, the concentration of several cytokines in sera was evaluated before and after immunization at various time points for subjects immunized with either dose of rBCG-N-hRSV or BCG-WT (**Supplementary Figure 3**). All subjects exhibited a slight increase in the concentrations of IFN-γ, IL-6, IL-10, and TNF-α in serum, which suggests the lack of severe adverse effects after immunization. However, further secretory cytokine profiling would be needed to confirm this observation. These results suggest that the doses of rBCG-N-hRSV tested do not induce peripheral inflammation, which supports the safety of the rBCG-N-hRSV vaccine candidate in healthy BCG-immunized adults.

### 3.3 rBCG-N-hRSV immunization induces the expansion of effector T cells

**Figure 3:**
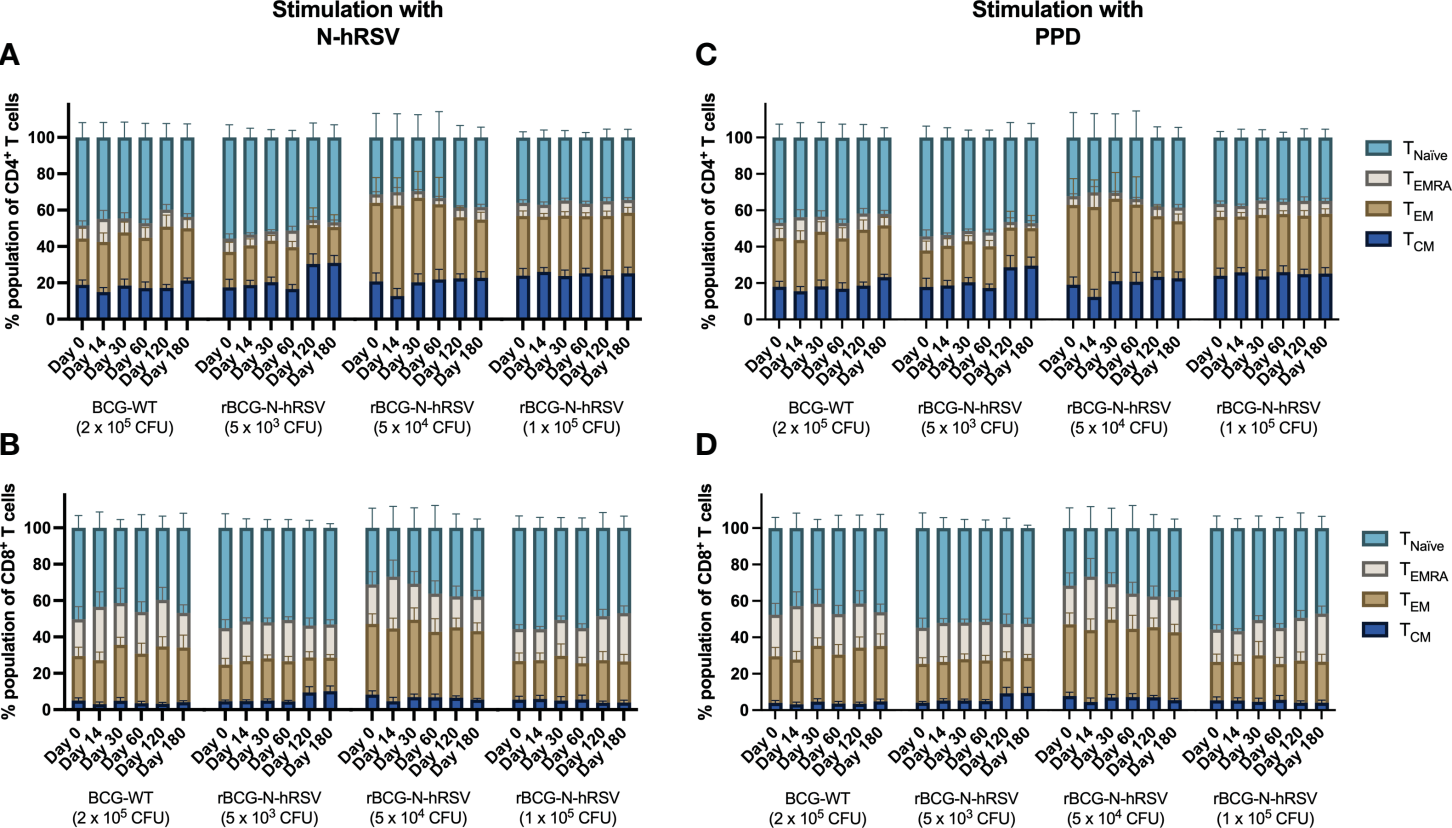
Expansion of T cell memory subsets after an immunization with a single dose of rBCG-N-hRSV. T cell subsets defined by the expression of CD62L and CD45RA were evaluated after stimulation of PBMCs with **(A, B)** N-hRSV or **(C, D)** PPD by flow cytometry. Stimulation of PBMCs with 5 µg/mL ConA or 0.5% sterile PBS were used as positive and negative stimulation controls, respectively (data not shown). T_Naïve_, T_EMRA_, T_EM_, and T_CM_ subsets are shown for **(A, C)** CD4^+^ T cells and **(B, D)** CD8^+^ T cells. Data for subjects potentially exposed to hRSV during the post-immunization period, as suggested by hRSV-specific serological assays, were excluded. Bars indicate means, while error bars represent SEM. A two-way ANOVA for repeated measures with *post-hoc* Dunnett's test corrected for multiple comparisons against Day 0 was performed for data analysis.

**Figure 4:**
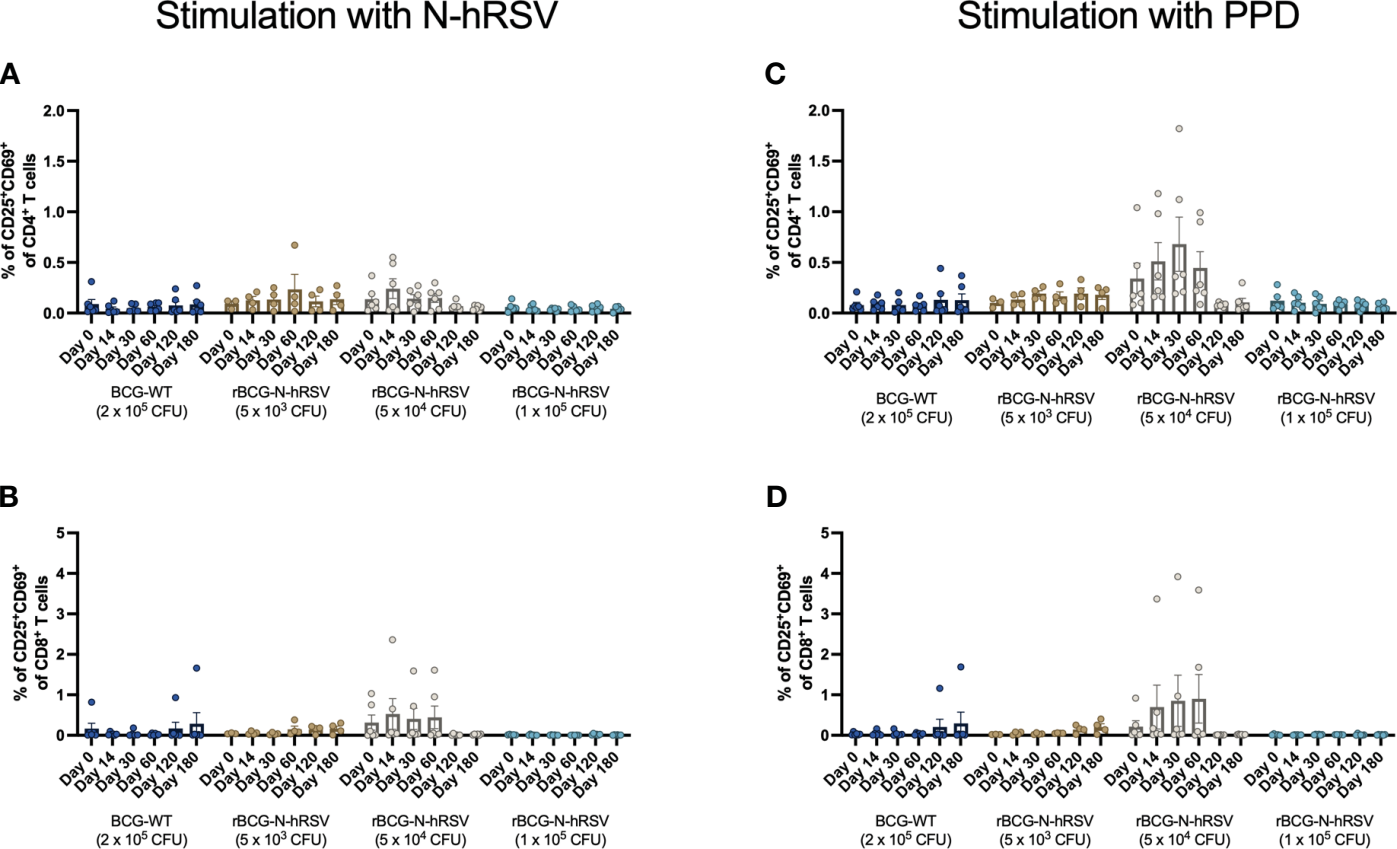
Activation of T cells in subjects immunized with rBCG-N-hRSV. Activation of T cells was assessed by flow cytometry. PBMCs were stimulated with either **(A, B)** N-hRSV or **(C, D)** PPD. Percentages of CD25^+^CD69^+^ **(A, C)** CD4^+^ T cell or **(B, D)** CD8^+^ T cell populations are shown. Stimulation of PBMCs with 5 µg/mL ConA or 0.5% sterile PBS were used as positive and negative stimulation controls, respectively (data not shown). Data for subjects potentially exposed to hRSV during the post-immunization period, as suggested by hRSV-specific serological assays, were excluded. Bars indicate means, error bars represent SEM. A two-way ANOVA for repeated measures with *post-hoc* Dunnett's test corrected for multiple comparisons against Day 0 was performed for data analysis.

**Figure 5:**
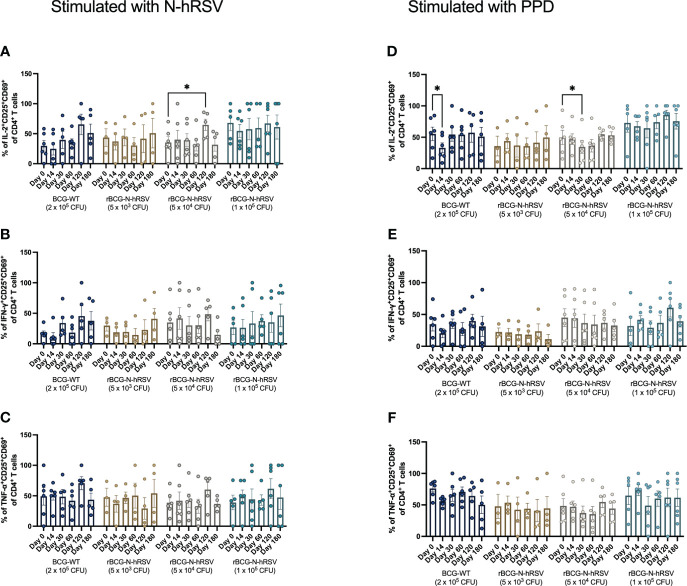
Cytokine production by activated CD4^+^ T cells from rBCG-N-hRSV immunized subjects. The profile of the activated CD4^+^ T cells was assessed by flow cytometry. PBMCs were stimulated with either **(A–C)** N-hRSV or **(D–F)** PPD. Percentages of IL-2^+^CD25^+^CD69^+^ in **(A, D)**, IFN-γ^+^CD25^+^CD69^+^ **(B, E)**, or TNF-α^+^CD25^+^CD69^+^ **(C, F)** CD4^+^ T cell populations are shown. Stimulation of PBMCs with 5 µg/mL ConA or 0.5% sterile PBS were used as positive and negative stimulation controls, respectively (data not shown). Data for subjects potentially exposed to hRSV during the post-immunization period, as suggested by hRSV-specific serological assays, were excluded. Bars indicate means, while error bars represent SEM. A two-way ANOVA for repeated measures with *post-hoc* Dunnett's test corrected for multiple comparisons against Day 0 was performed for data analysis. * = P<0.05.

**Figure 6:**
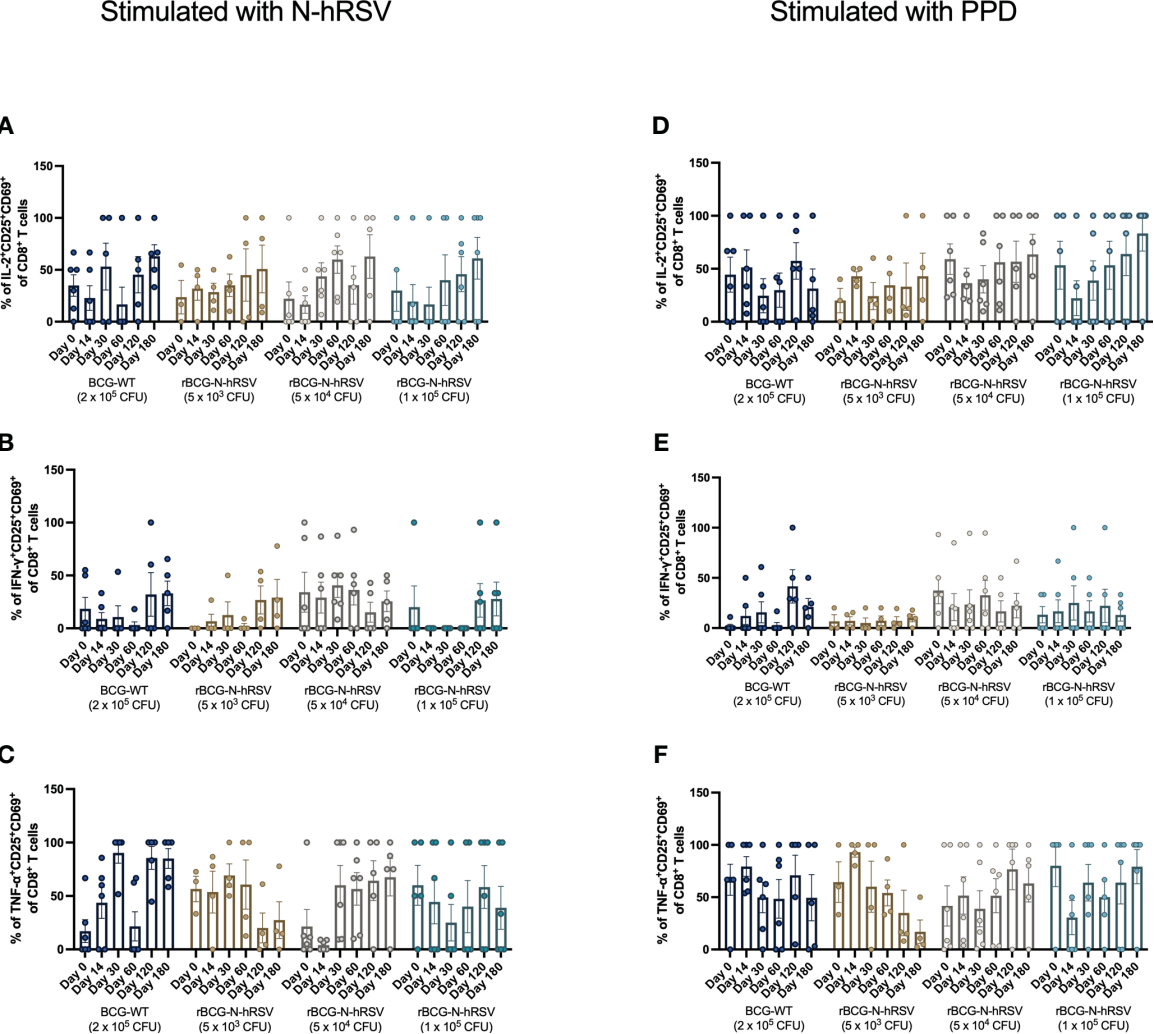
Cytokine production by activated CD8^+^ T cells from rBCG-N-hRSV immunized subjects. The profile of activated CD8^+^ T cells was assessed by flow cytometry. PBMCs were stimulated with either **(A–C)** N-hRSV or **(D–F)** PPD. Percentages of IL-2^+^CD25^+^CD69^+^ in **(A, D)**, IFN-γ^+^CD25^+^CD69^+^ **(B, E)**, or TNF-α^+^CD25^+^CD69^+^ **(C, F)** CD8^+^ T cell populations are shown. Stimulation of PBMCs with 5 µg/mL ConA or 0.5% sterile PBS were used as positive and negative stimulation controls, respectively (data not shown). Data for subjects potentially exposed to hRSV during the post-immunization period, as suggested by hRSV-specific serological assays, were excluded. Bars indicate means, while error bars represent SEM. A two-way ANOVA for repeated measures with *post-hoc* Dunnett's test corrected for multiple comparisons against Day 0 was performed for data analysis.

Since we detected an antigen-specific T cell response in subjects immunized with rBCG-N-hRSV, we sought to determine whether immunization promotes the expansion of memory T cell subsets (gating on **Supplementary Figure 4**) (23). Albeit non-statistically significant, stimulation of PBMCs with N-hRSV or PPD led to a contraction of the naïve population along with the expansion of the effector memory (T_EM_) and the CD45RA-expressing effector memory (T_EMRA_) CD4^+^ T cell populations in participants immunized with 5x10^4^ CFU of rBCG-N-hRSV 14 days post-immunization (**Figure 3**). Interestingly, similar contractions of the naïve population and the expansion of T_EM_ and T_EMRA_ was detected in subjects immunized with 1x10^5^ CFU of rBCG-N-hRSV, with an even tighter deviation relative to the 5x10^4^ CFU group (**Figure 3**). This was also accompanied by a minor increase in the percentage of PPD-stimulated CD4^+^ T cells co-expressing CD25 and CD69 (**Figure 4**). However, the significant variability between subjects needs to be considered. Interestingly, a significant increase in the percentage of N-hRSV-stimulated CD4^+^ T cells expressing IL-2 could be observed in these participants at day 120 post-immunization in subjects immunized with 5x10^4^ CFU of rBCG-N-hRSV (**Figure 5**). A slight increase of IFN-γ and TNF-α expression, when stimulated with either PPD or N-hRSV, can be observed in this cohort at day 120 post-immunization (**Figure 5**). This increase at day 120 post-immunization is similar to the increase observed with BCG-WT, but not with 5x10^3^ and 1x10^5^ CFU of rBCG-N-hRSV. A mild increase in CD69-expressing CD4^+^ T cells was also observed for PBMCs from participants immunized with the lowest dose of rBCG when stimulated with N-hRSV (**Supplementary Figure 5**). A slight increase of N-hRSV- or PPD-stimulated CD8^+^ T cells expressing IL-2 or IFN-γ in participants immunized with 5x10^4^ or 1x10^5^ CFU of rBCG-N-hRSV was detected (**Figure 6**).

### 3.4 rBCG-N-hRSV induced hRSV- and mycobacterial-specific antibodies with reduced C1q-binding capacity as compared to basal-level antibodies

Although the concentration of anti-N-hRSV antibodies in the serum only significantly changed after immunization with 1x10^5^ CFU of rBCG-N-hRSV (**Supplementary Figure 6**) (9), it is relevant to evaluate the relative proportions of anti-N-hRSV IgG subclasses in the samples of all subjects and the potential deposition of complement proteins mediated by these antibodies to rule out potential lung damage that could be driven by anti-N IgG in the absence of a robust neutralizing response in infants (17). Since lung damage has been associated with excessive complement system activation, the ability of anti-N-hRSV antibodies to bind exogenously-added C1q was determined as a functional measurement for the induced anti-N-hRSV IgG subclasses (24).

**Figure 7:**
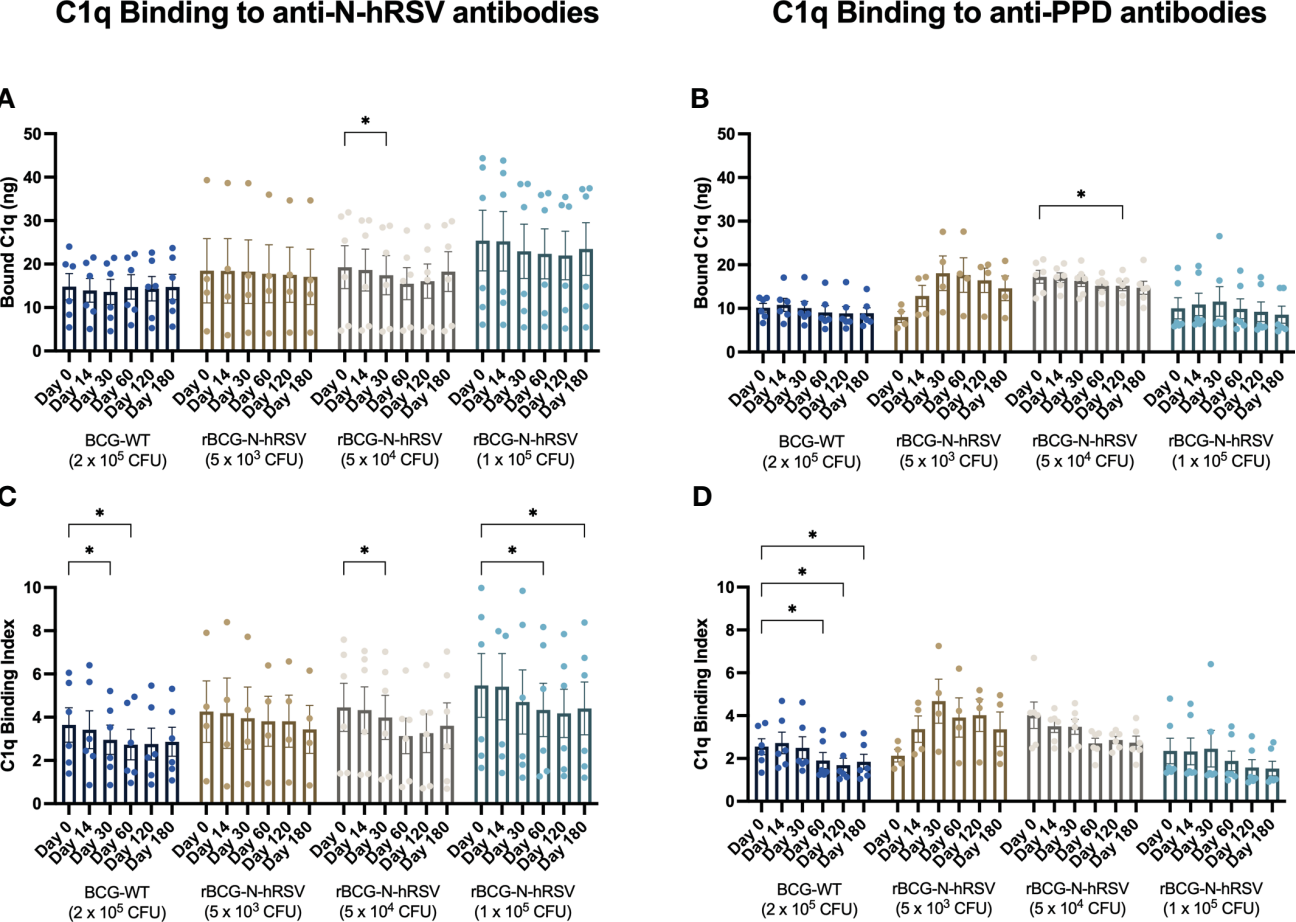
N-hRSV- and anti-PPD-specific serum antibodies from rBCG-N-hRSV immunized subjects show reduced binding of C1q. Total C1q binding is shown for **(A)** total anti-N-hRSV antibodies and **(B)** total anti-PPD antibodies. A C1q Binding Index was calculated by dividing total bound C1q by the log_10_ of total IgG against **(C)** N-hRSV or **(D)** PPD. Data for subjects potentially exposed to hRSV during the post-immunization period, as suggested by hRSV-specific serological assays, were excluded. Bars indicate means, while error bars represent SEM. A two-way ANOVA for repeated measures with *post-hoc* Dunnett's test corrected for multiple comparisons against Day 0 was performed for data analysis. * = P<0.05.

Exogenous C1q binding to anti-N-hRSV or anti-PPD antibodies in sera was quantified for each serum sample (**Figure 7**). Both anti-N-hRSV and anti-PPD antibodies from subjects immunized with 5x10^4^ and 1x10^5^ CFU of rBCG-N-hRSV were found to bind similar, or less complement after immunization as compared to day 0 post-immunization, which was the day with highest binding to IgG. These trends were maintained when C1q binding was normalized by total anti-N or anti-PPD IgG titers (C1q Binding Index). However, C1q binding for both anti-N-hRSV and anti-PPD antibodies from subjects immunized with 5x10^3^ CFU of rBCG-N-hRSV increased after immunization. Fold changes compared to the pre-immune condition showed similar trends (**Supplementary Figure 7**).

**Figure 8:**
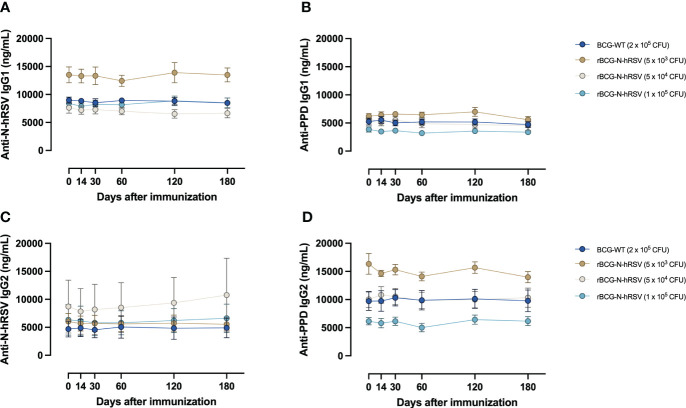
Immunization with rBCG-N-hRSV does not induce differential secretion of anti-N-hRSV or anti-PPD IgG1 and IgG2 antibodies. **(A, B)** IgG1 and **(C, D)** IgG2 antibody titers were measured using ELISA. Depicted are **(A, C)** anti-N-hRSV and **(B, D)** anti-PPD antibody concentrations over time. Data for subjects potentially exposed to hRSV during the post-immunization period, as suggested by hRSV-specific serological assays, were excluded. Dots represent mean titers, while error bars represent SEM. A two-way ANOVA for repeated measures with *post-hoc* Dunnett's test corrected for multiple comparisons against Day 0 was performed for analysis of data.

Although the amount of C1q binding to purified antigen-specific IgG subclasses was not evaluated, we did evaluate antigen-specific IgG isotypes in sera that bound to N-hRSV or PPD as a proxy of this function. Interestingly, immunization with rBCG-N-hRSV did not change the concentration of IgG subclasses, neither for anti-N-hRSV nor for anti-PPD antibodies (**Figure 8**). As expected, IgG1 titers for anti-N-hRSV were higher than IgG2 titers. To further confirm that there was no induction of potentially harmful, non-neutralizing, and highly complement-binding anti-N-hRSV IgG, we measured anti-N-hRSV IgG3 levels. As expected, we found no quantifiable antigen-specific IgG3 signals, further supporting the lack of harmful complement-binding responses (**Supplementary Figure 8**).

### 3.5 Immunization with the lowest rBCG-N-hRSV dose and potential hRSV exposure led to mixed immune responses

Although participants reported no symptoms of a common cold during the study, two of them presented serological evidence of hRSV exposure (>two-fold increase in anti-F-hRSV and anti-G-hRSV IgG titers) (**Supplementary Table 1**, **Supplementary Figure 9**). Both subjects were part of the cohort immunized with the lowest tested dose of rBCG-N-hRSV, and their data were excluded from the global analysis of the study.

One participant was probably exposed to hRSV between 14 and 30 dpi and exhibited increased serum cytokines, slightly increased C1q binding to anti-N-hRSV antibodies, no increase in anti-N-hRSV IgG subclasses, increased Perf, GrzB, TNF-α, and IFN-γ secretion by PBMCs upon N-hRSV stimulation, and no changes in T cell memory phenotypes or expression of activation markers by 30 dpi (**Supplementary Figure 9**). This participant also showed modest cellular responses to PPD stimulation, marked by delayed Perf and GrzB secretion and early TNF-α and IFN-γ secretion by PBMCs (**Supplementary Figure 10**).

The other participant was probably exposed before 14 dpi and showed no systemic cytokine responses, a slight induction of C1q binding to anti-N-hRSV antibodies and anti-N-hRSV IgG1, no Perf or GrzB responses to N-hRSV or changes in memory phenotypes, increased activation of CD8^+^ T cells (as measured by the expansion of the CD69-expressing population), and a marked increase in the secretion of cytokines by PBMCs stimulated with N-hRSV (**Supplementary Figure 9**). This subject also showed poor responsiveness to PPD, characterized only by the secretion of cytokines by PBMCs, but no Perf or GrzB responses (**Supplementary Figure 10**).

## 4 Discussion

Cytokine concentrations measured in sera constitute a good immunological indicator of the safety of rBCG-N-hRSV (**Supplementary Figure 3**). A slight tendency toward increased IFN-γ was observed for all experimental groups, except for subjects immunized with the lowest dose of rBCG-N-hRSV. The increase in IFN-γ concentration is considered a positive result since this short-lived response may indicate that immunization with higher doses of WT, or a recombinant BCG induces generalized T_H_1 responses (10, 25), and it is comparable to the positive control (ConA) (Data not shown) (23, 24). This was expected as BCG is a known promoter of T_H_1 responses, which is the most effective response against intracellular pathogens, such as hRSV (26). Additionally, because the infection induced by hRSV elicits an immune profile different from T_H_1, and due to the fact that BCG is able to induce this profile, this bacterium was selected as a platform for the antigenic delivery of the N-hRSV protein (26).

rBCG-N-hRSV was shown to promote a T_H_1 polarization of the immune response against N-hRSV in healthy adults, as measured by secretion of IFN-γ by PBMCs (9). Our results further support this since subjects immunized with 5x10^4^ CFU of rBCG-N-hRSV showed a considerable perforin and granzyme B response against PPD and N-hRSV (**Figure 1**), as well as the increase in IFN-γ concentration in PBMC cultures (**Figure 2**). The secretion of these cytotoxic molecules indicates that upon a natural infection with hRSV in these subjects, vaccination with rBCG-N-hRSV at this dose might be sufficient to confer protection against disease. An unresponsiveness in the secretion of cytotoxic molecules by PBMCs from subjects immunized with the lowest and highest doses of rBCG-N-hRSV in response to N-hRSV could be explained by insufficient antigen delivery and N-hRSV masking by mycobacterial antigens, respectively.

Although we did not detect a significant expansion of memory T cells or activated T cells upon immunization with BCG-WT or rBCG-N-hRSV (**Figures 3**, **4**), we did detect a significant expression of IL-2 only in activated CD4^+^ T cells stimulated with N-hRSV at 120 days after immunization (**Figures 5**, **6**). Natural infection with hRSV after immunization could enhance the expansion of memory T cell subsets, as seen for other viruses (27, 28). This was not detected for the subjects suspected to be exposed to hRSV after immunization (**Supplementary Figure 9**), and it may be due to an insufficient vaccine dose, the limited time between vaccination and virus exposure, or pre-existing memory due to prior priming with the virus.

Decreased complement binding via the classical pathway was observed after immunization with rBCG-N-hRSV, especially for the higher doses tested. However, no detectable changes in concentrations of IgG1 or IgG2 specific against N-hRSV or PPD were found. We found no detectable anti-N-hRSV IgG3 in sera, consistent with a lack of increased C1q binding. The relevance of this subtle change in antibody effector function remains to be determined but is likely a good indicator of the safety of this vaccine (29).

Increased anti-F-hRSV and anti-G-hRSV antibody titers were detected for two subjects. They reported no symptoms of hRSV infection, so an asymptomatic infection was probably developed (30). While their immune responses against N-hRSV were mixed, so were their responses to PPD. Possibly, their responsiveness to PPD was correlated with responsiveness to N-hRSV, given the low dose of vaccine administered. Also, considering that these possible breakthrough infections were asymptomatic, this further supports that this vaccine could protect against this virus.

The main limitations of this study include a low sample size, as the primary outcome of the clinical trial was to evaluate safety. One possible confounding variable is the exposure to hRSV during the study. In this regard, it could be beneficial to conduct shorter clinical studies between hRSV seasons or with quarantined participants to reduce hRSV exposure after vaccination to assess the immune response induced by the vaccine. The immune response evaluated herein was assessed only during the immune steady state of pre-primed adults, so differential immune responses after infection are yet to be determined. Another possible variable is that the healthy adults participating in this study were previously immunized with BCG. It would be relevant to test the rBCG-N-hRSV vaccine in a population that has not been previously immunized with BCG, as this vaccine candidate is intended to be used instead of the standard BCG vaccine upon birth. Lastly, while evaluation of the immune response elicited in healthy male adults is necessary before testing this vaccine in the pediatric population, the response elicited in newborns and infants is far more relevant, considering that they are the target population for immunization. Altogether, these results support that the vaccine is safe and immunogenic and suggest that the implementation of a phase II clinical trial would be the next appropriate step for the development of this vaccine.

## 5 Conclusions

hRSV is the major viral pathogen responsible for acute lower respiratory tract infections in children. This virus causes millions of hospitalizations and a significant number of deaths yearly. Despite the substantial social and economic burden that hRSV poses, no licensed hRSV vaccines are available to date for the pediatric population, although several candidates are being developed. In Chile, a phase I clinical trial (NCT03213405) was held to evaluate the safety, tolerability, and shedding of this rBCG-N-hRSV vaccine candidate in healthy male adults. Even though an initial evaluation of the rBCG-N-hRSV vaccine candidate was performed during a phase I clinical trial, further evaluations support advancement into a phase II trial. Indeed, a deeper immunogenicity analysis will provide more information regarding the rBCG-N-hRSV vaccine candidate. These results support that rBCG-N-hRSV is safe and immunogenic un adults. This vaccine candidate induces a cellular immune response upon stimulation with viral and mycobacterial antigens, which is comparable to the positive control (ConA). These results contribute to pursuing the development of phase II clinical trials, which are currently being prepared.

## Data availability statement

The original contributions presented in the study are included in the article/**Supplementary Material**. Further inquiries can be directed to the corresponding author.

## Ethics statement

The studies involving human participants were reviewed and approved by Institutional Ethical Committee (number 15216) and the Chilean Public Health Institute (ISP Chile, number EC819077/16). The patients/participants provided their written informed consent to participate in this study.

## Author contributions

Conceptualization: GP, CA, NG, PG, SB, AK. Visualization: GP, NG, CA, PG, SB, AK. Methodology: GP, CA, NG, LR-G, YV. Investigation: GP, CA, NG, LR-G, YV, PG, SB, AK. Funding acquisition: PG, SB, AK. Project administration: PG, SB, AK. Supervision: PG, SB, AK. Writing – original draft: GP, AK. Writing – Review and editing: GP, NG, CA, LR-G, YV, PG, SB, AK, KA. All authors contributed to the article and approved the submitted version.
