# Supplementary material for: Characterization of the humoral and cellular immunity induced by a recombinant BCG vaccine for the respiratory syncytial virus in healthy adults
Source: Front Immunol. 2023 Jul 18;14:1215893. doi: 10.3389/fimmu.2023.1215893 (PMC10390696; doi:10.3389/fimmu.2023.1215893)
Supplement: Supplementary file 1 [file DataSheet_1.docx]

Supplementary Material

Characterization of the humoral and cellular immunity induced by a recombinant BCG vaccine for the respiratory syncytial virus in healthy adults

Gaspar A. Pacheco ^1†^, Catalina A. Andrade ^1†^, Nicolás M. S. Gálvez ^1^, Yaneisi Vázquez ^1^, Linmar Rodríguez-Guilarte ^1^, Katia Abarca ^1,2^, Pablo A. González ^1^, Susan M. Bueno ^1^, Alexis M. Kalergis ^1,3*^

*** Correspondence:**Dr. Alexis M. Kalergis

Full Professor, Pontificia Universidad Católica de Chile

Director, Millennium Institute of Immunology and Immunotherapy

Av. Libertador Bernardo O’Higgins 340

Email: [akalergis@bio.puc.cl](mailto:akalergis@bio.puc.cl).

1. **Supplementary table**

**Table 1. Seroconversion analyses to determine possible exposure to hRSV from vaccinated volunteers.**

1. **Supplementary figures**


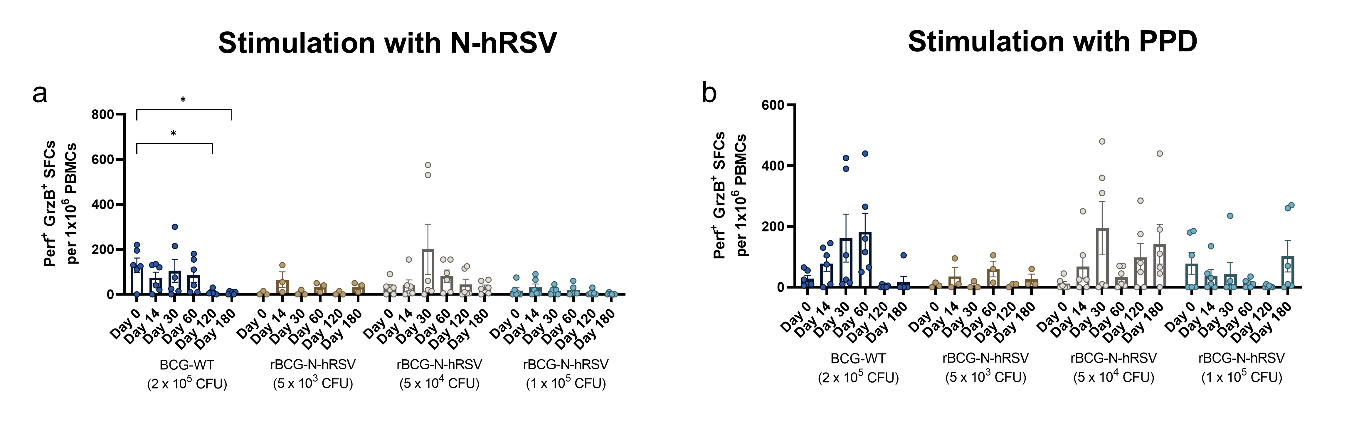


**Supplementary Figure 1. Perf and GrzB double-positive cells measured by ELISPOT.** Perf^+^ GrzB^+^ spot forming cells (SFCs) were counted after PBMCs were stimulated for 48 h with either 1.25 µg/mL N-hRSV **(a)** or 750 IU/mL PPD **(b)**. Bars represent the mean value of SFCs, and error bars represent the SEM. A two-way ANOVA for repeated measures with *post-hoc* Dunnet’s test corrected for multiple comparisons against Day 0 was performed for analysis of data. * = p<0.05.


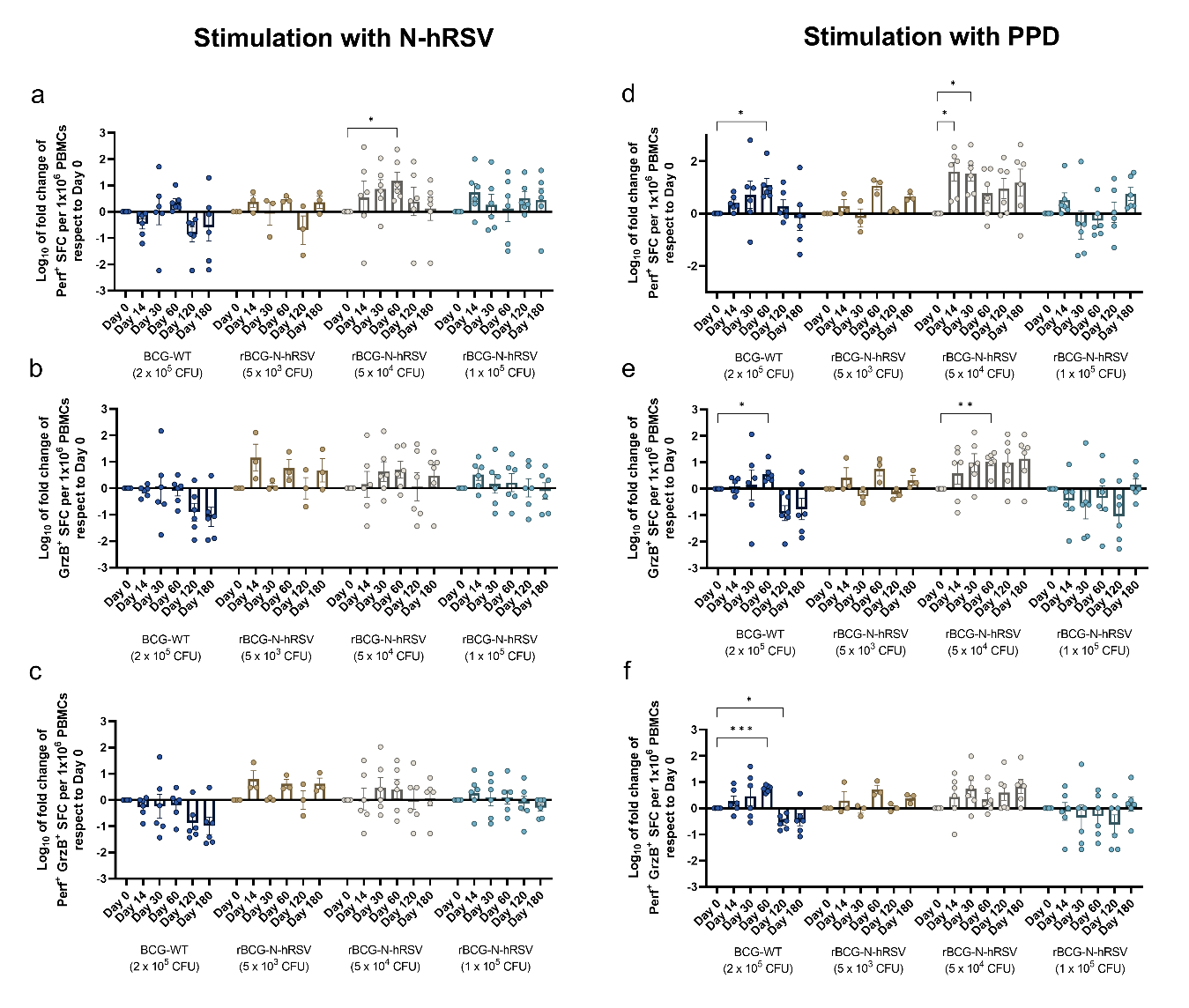


**Supplementary Figure 2. Fold changes of Perf^+^ and GrzB^+^ spot forming cells (SFCs).** Base 10 logarithms of fold changes of **(a, d)** Perf^+^, **(b, e)** GrzB^+^, or **(c, f)** Perf^+^ GrzB^+^ SFCs relative to day 0 are shown. PBMCs were stimulated for 48 h with either 1.25 µg/mL N-hRSV **(a-c)** or 750 IU/mL PPD **(d-f)**. Bars represent the mean value of SFCs, and error bars represent the SEM. A two-way ANOVA for repeated measures with *post-hoc* Dunnet’s test corrected for multiple comparisons against Day 0 was performed for analysis of data. * = p<0.05, ** = p<0.01.


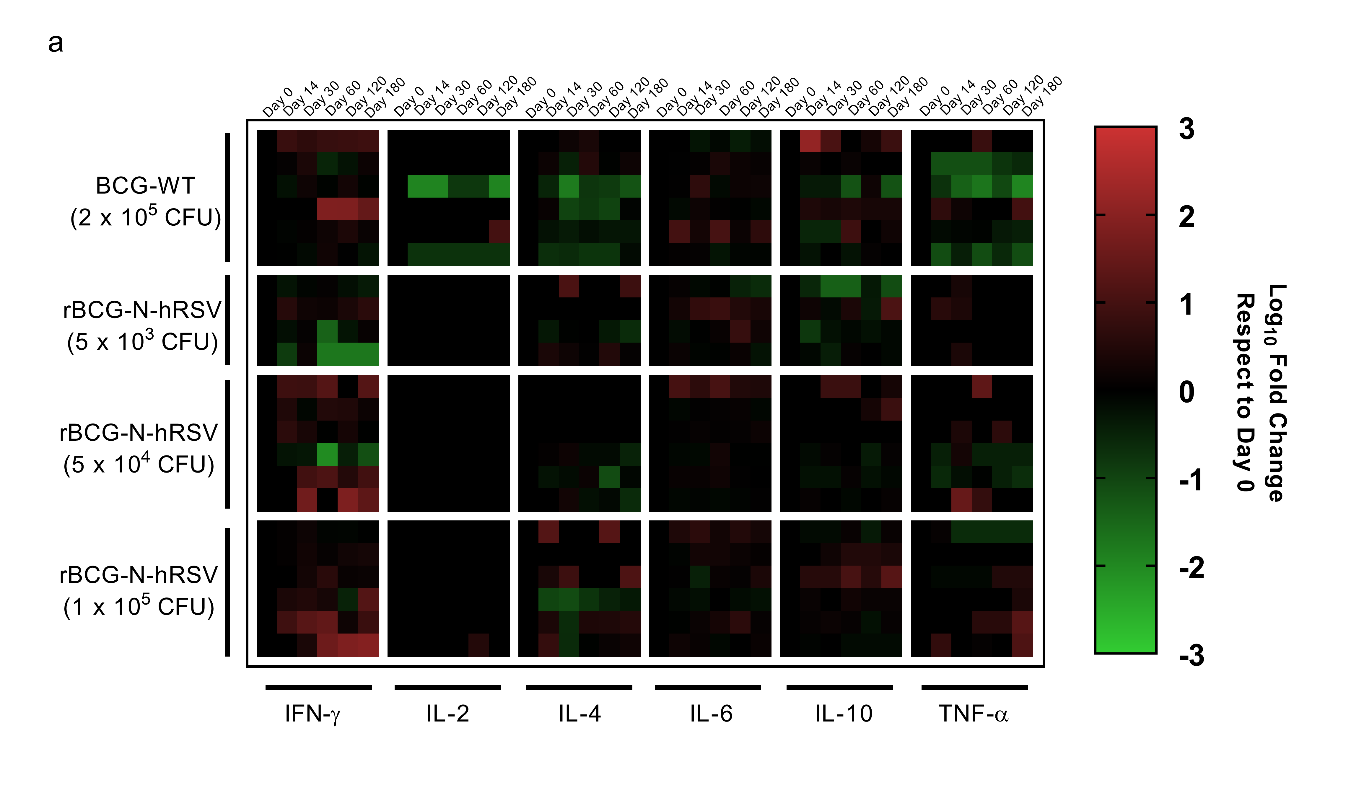


**Supplementary Figure 3. Immunization with rBCG-N-hRSV leads to a limited increase in serum cytokine concentrations.** Heatmap of log_10_ fold change of the concentration of the cytokines IFN-γ, IL-2, IL-4, IL-6, IL-10, and TNF-α in sera samples relative to day 0. Each block of columns represents a particular cytokine, labeled below. Individual columns represent timepoints after immunization, specified above. Each block of rows represents a particular cohort of immunized study subjects, labeled left. Individual rows represent individual subjects. A color scale is depicted on the right. Data for subjects potentially exposed to hRSV during the post-immunization period, as suggested by hRSV-specific serological assays, were excluded.


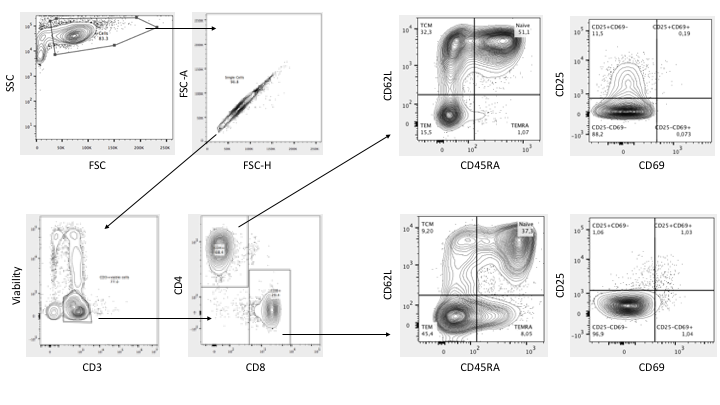


**Supplementary Figure 4. Gating strategy for the identification of T cell subsets.** T cell subsets defined by the expression of CCR7 and CD45RA were evaluated after stimulation of PBMCs with N-hRSV or PPD. CD4^+^ or CD8^+^ T cell populations were identified as naïve (CD62L^+^ CD45RA^+^), central memory (T_CM_, CD62L^+^ CD45RA^-^), effector memory (T_EM_, CD62L^-^ CD45RA^-^), or CD45RA-expressing effector memory (T_EMRA_, CD62L^-^ CD45RA^+^). Additionally, activation markers CD25 and CD69 were incorporated in the gating strategy for CD4^+^ and CD8^+^ T cell populations to evaluate expansion of activated populations in response to antigen.


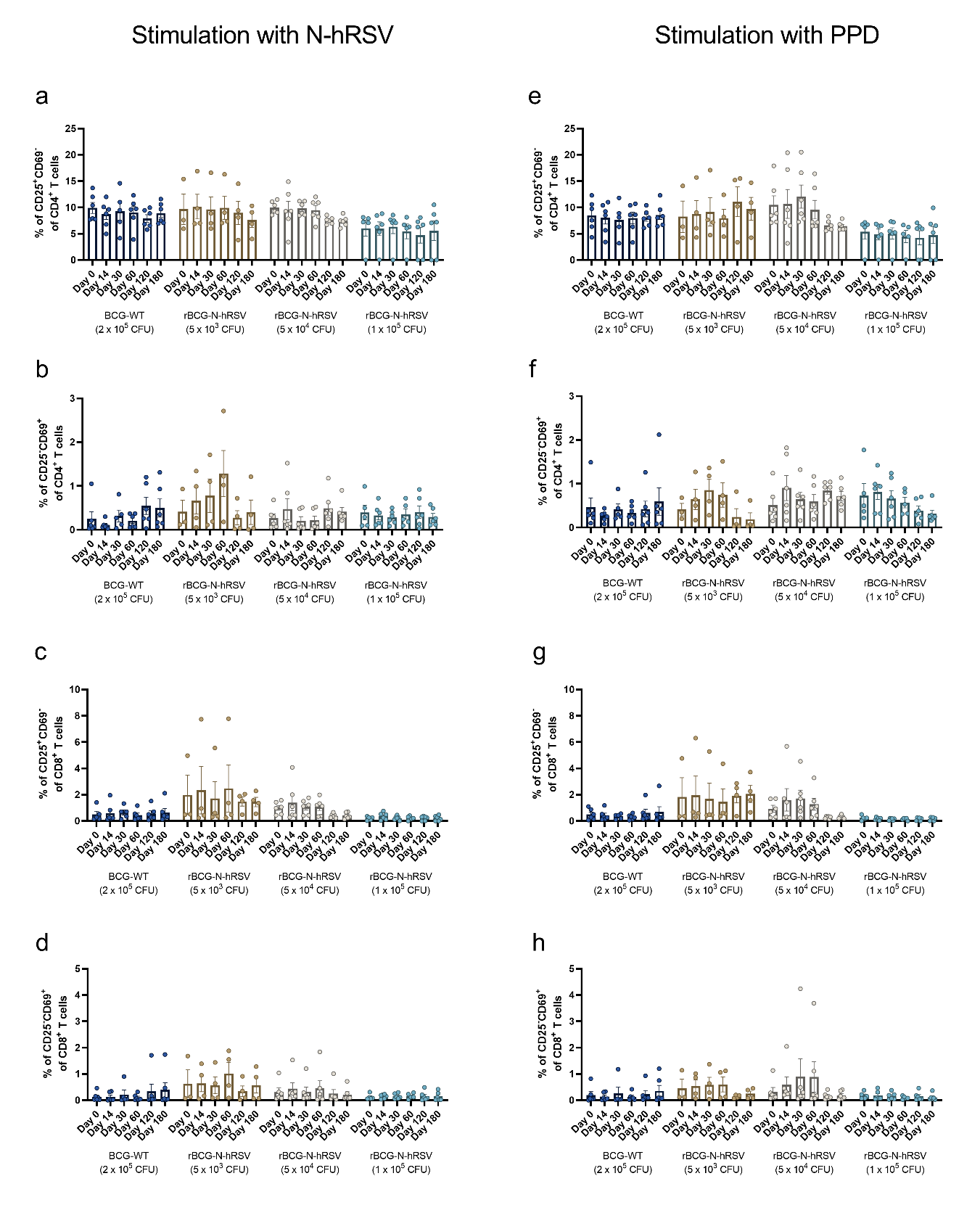


**Supplementary Figure 5. Activation of T cell populations in subjects immunized with rBCG-N-hRSV.** Activation of T cells was assessed by flow cytometry. PBMCs were stimulated with either **(a-d)** N-hRSV or **(e-h)** PPD. Percentages of CD25^+^CD69^-^ **(a-b)** CD4^+^ T or **(e-f)** CD8^+^ T cell populations are shown. Additionally, percentages of CD25^-^CD69^+^ **(c-d)** CD4^+^ T or **(g-h)** CD8^+^ T cell populations are shown. Stimulation of PBMCs with 5 µg/mL ConA or 0.5% sterile PBS were used as positive and negative stimulation controls, respectively (data not shown). Data for subjects potentially exposed to hRSV during the post-immunization period, as suggested by RSV-specific serological assays, were excluded. Bars indicate means, while error bars represent SEM. A two-way ANOVA for repeated measures with *post-hoc* Dunnet’s test corrected for multiple comparisons relative to Day 0 was performed for data analysis. No statistical significant differences were found.


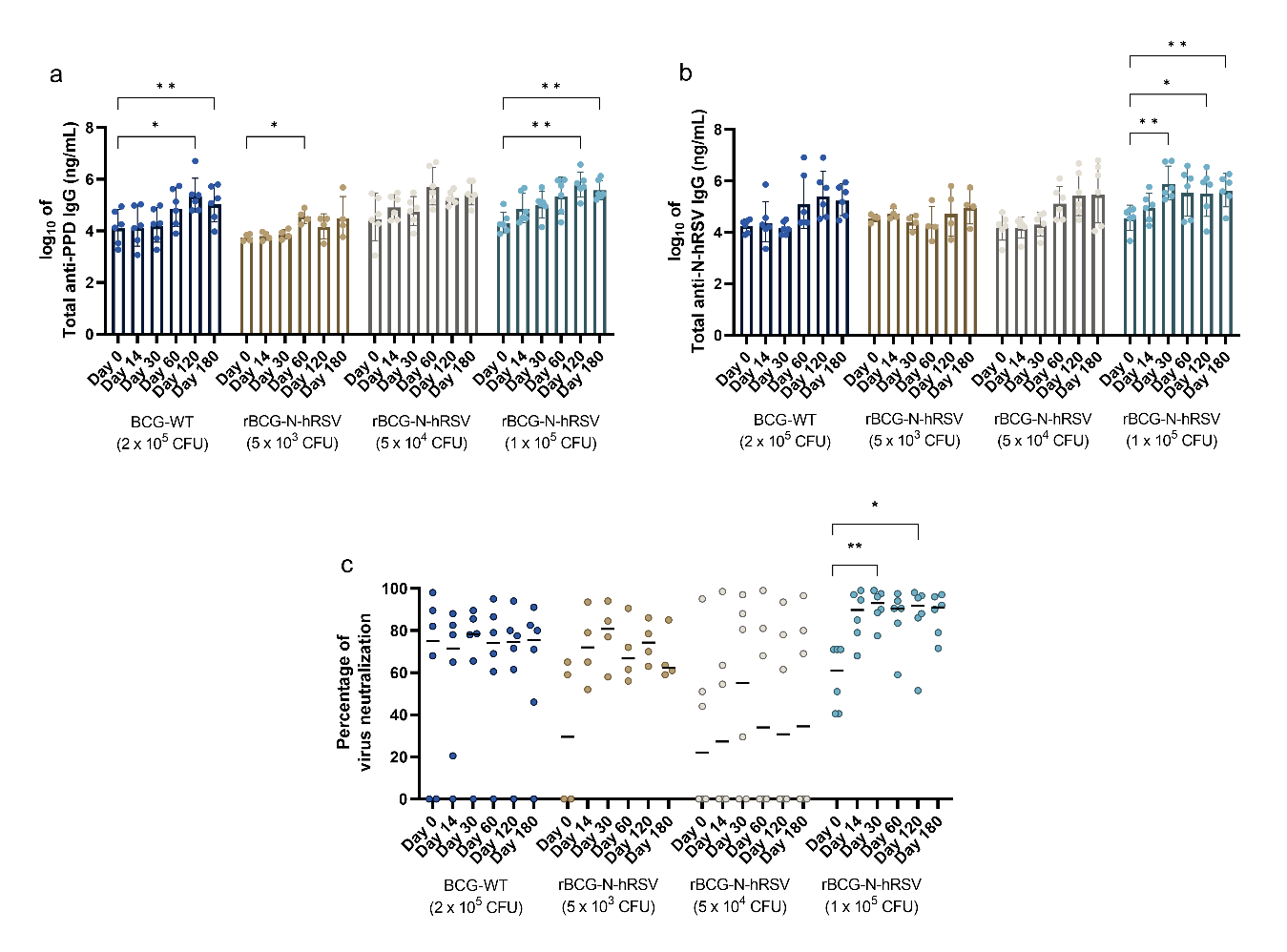


**Supplementary Figure 6. Total anti-PPD, anti-N-hRSV and neutralizing antibody concentrations in subjects immunized with rBCG-N-hRSV.** Base 10 logarithms of **(a)** total anti-PPD and **(b)** total anti-N-hRSV antibody concentrations in serum is shown. Bars indicate the mean and error bars indicate SEM. A two-way ANOVA for repeated measures with *post-hoc* Dunnet’s test corrected for multiple comparisons against Day 0 was performed for analysis of data. **(c)** The percentage of virus neutralization relative to a negative neutralization control after incubation of virus with diluted sera is shown. Bars indicate medians. A non-parametric Friedman test for repeated measures with *a posteriori* Dunn’s multiple comparisons between ranks of every column and the rank of Day 0 was performed individually for each cohort. * = p<0.05, ** = p<0.01. This data has been published by Abarca *et al.* (2020) (doi: 10.1016/j.eclinm.2020.100517) and is shown for reference.


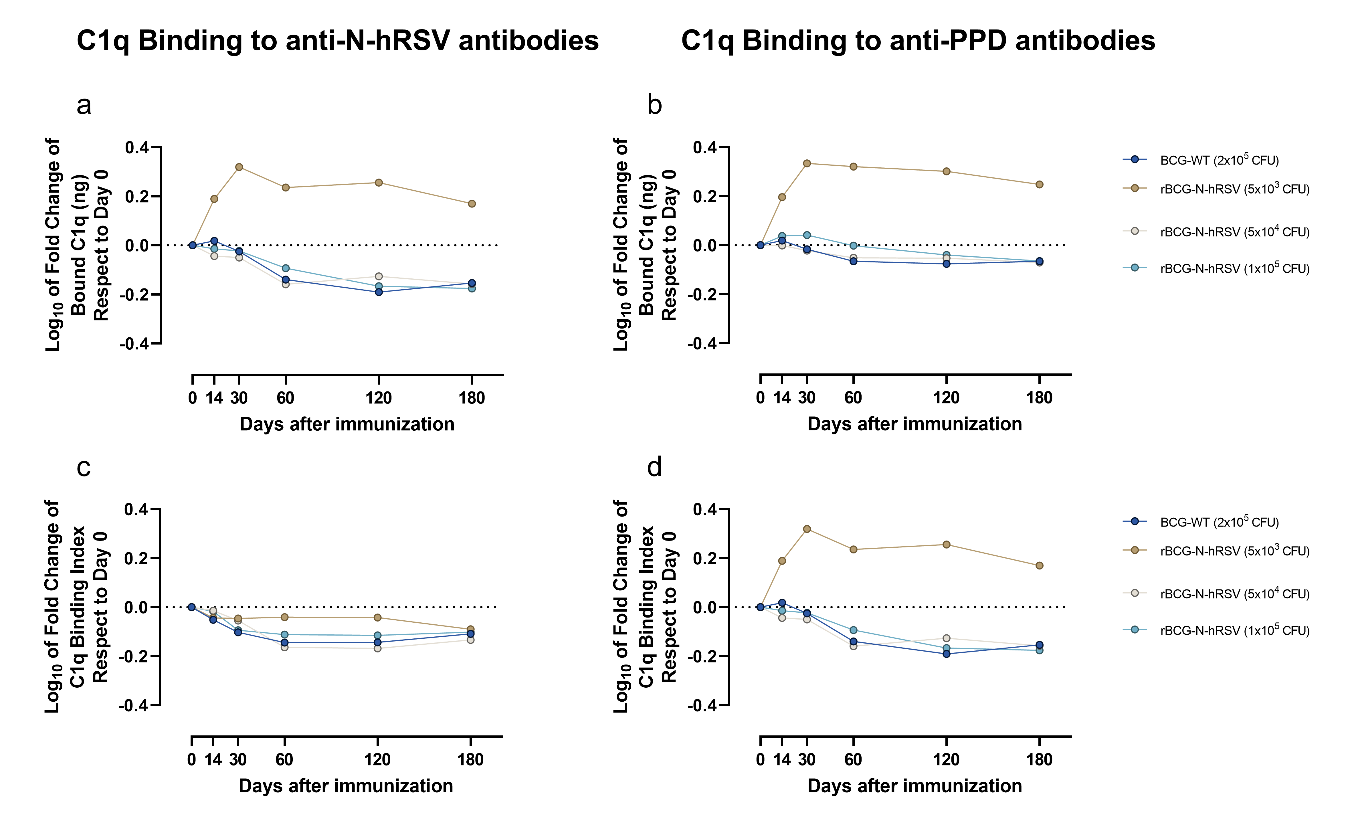


**Supplementary Figure 7. Fold changes of C1q binding by antibodies elicited after immunization with rBCG-N-hRSV.** Base 10 logarithms of fold changes of **(a, b)** total C1q binding and **(c, d)** C1q binding index is shown for **(a, c)** total anti-N-hRSV antibodies and **(b, d)** total anti-PPD antibodies. Bars indicate means. A two-way ANOVA for repeated measures with *post-hoc* Dunnet’s test corrected for multiple comparisons relative to Day 0 was performed for the analysis of the data.


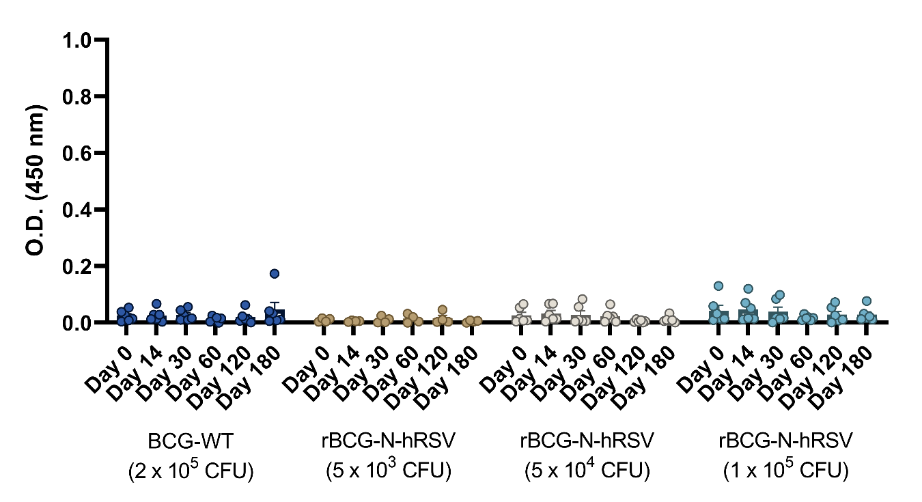


**Supplementary Figure 8. Immunization with rBCG-N-hRSV does not induce anti-N-hRSV IgG3.** Optical density at 450 nm in ELISA assays detecting anti-N-hRSV IgG3 present in the sera of the subjects. Bars indicate means and error bars represent SEM. A two-way ANOVA for repeated measures with *post-hoc* Dunnet’s test corrected for multiple comparisons relative to Day 0 was performed for the analysis of the data.


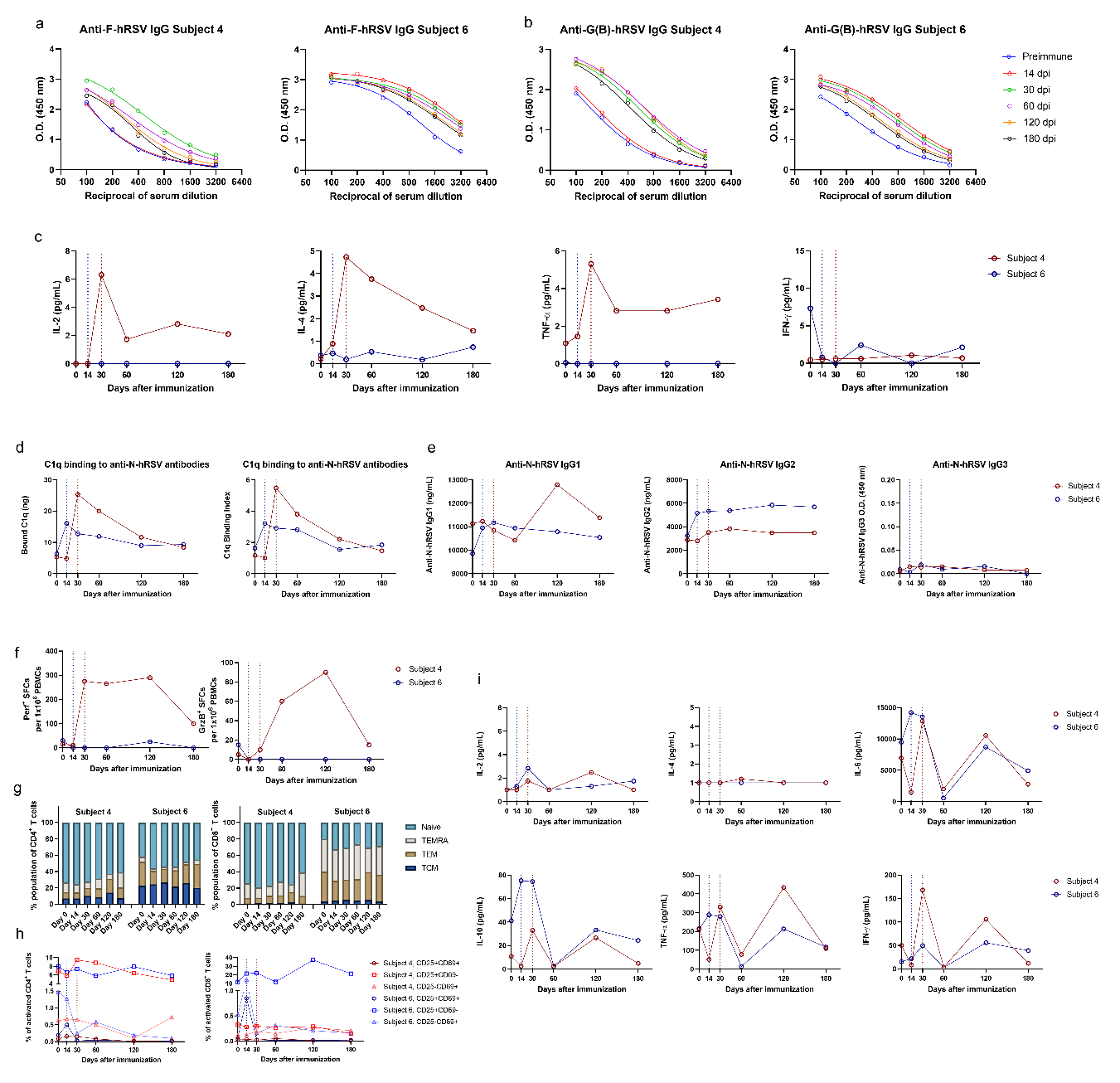


**Supplementary Figure 9. Immune response against N-hRSV in subjects immunized with 5x10^3^ CFU of rBCG-N-hRSV that were potentially exposed to hRSV infection.** Serum dilution curves for **(a)** anti-F-hRSV and **(b)** anti-G(B)-hRSV IgG antibodies are shown. **(c)** Cytokine concentrations in serum were determined using CBA^TM^. **(d)** C1q binding to anti-N-hRSV antibodies and **(e)** anti-N-hRSV IgG subclasses were measured using ELISA. PBMC cultures were stimulated with N-hRSV and **(f)** Perf and GrzB spot-forming cells were counted by ELISPOT. **(g)** Memory and **(h)** activated CD4^+^ and CD8^+^ T cell populations were assessed via flow cytometry, and **(i)** the concentration of cytokines was measured by CBA^TM^ in culture supernatants. Colored dotted vertical lines in **c, d, e, f, h, and i** correspond to the timepoints in which anti-F-hRSV and anti-G(B)-hRSV antibody titers were considered serological evidence of potential hRSV exposure.


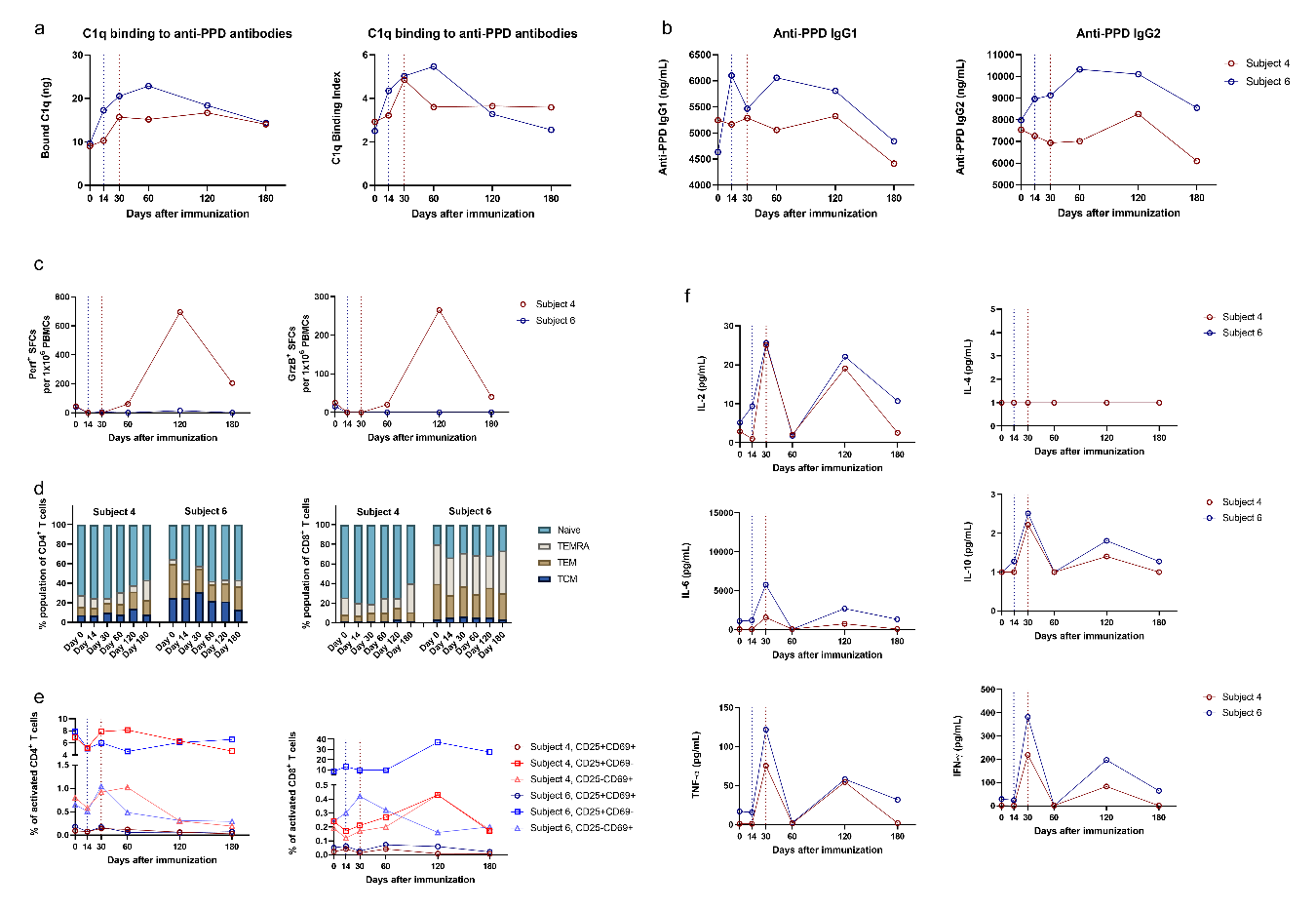


**Supplementary Figure 10. Humoral and cellular responses to PPD in subjects with breakthrough asymptomatic hRSV infections. (a)** C1q binding to anti-PPD antibodies and **(b)** anti-PPD IgG subclasses were measured using ELISA. PBMC cultures were stimulated with PPD and **(c)** Perf and GrzB spot-forming cells were counted using ELISPOT. **(d)** Memory and **(e)** activated CD4^+^ and CD8^+^ T cell populations were assessed by flow cytometry, and **(f)** the concentration of cytokines was measured by CBA^TM^ in culture supernatants. Colored dotted vertical lines in **a, b, c, e, and f** correspond to the timepoints in which anti-F-hRSV and anti-G(B)-hRSV antibody titers were considered serological evidence of hRSV exposure.

**REFERENCE**

1. World Medical Association. World Medical Association Declaration of Helsinki: ethical principles for medical research involving human subjects. JAMA. 2013 Nov;310(20):2191–4.

2. Abarca K, Rey-Jurado E, Muñoz-Durango N, Vázquez Y, Soto JA, Gálvez NMS, et al. Safety and immunogenicity evaluation of recombinant BCG vaccine against respiratory syncytial virus in a randomized, double-blind, placebo-controlled phase I clinical trial. EClinicalMedicine. 2020 Oct;27:100517.
